# Accommodation is unrelated to myopia progression in Chinese myopic children

**DOI:** 10.1038/s41598-020-68859-6

**Published:** 2020-07-21

**Authors:** Yunyun Chen, Björn Drobe, Chuanchuan Zhang, Nisha Singh, Daniel P. Spiegel, Hao Chen, Jinhua Bao, Fan Lu

**Affiliations:** 1 School of Ophthalmology and Optometry, Wenzhou Medical University, Wenzhou, China; 2 WEIRC, WMU-Essilor International Research Center, Wenzhou, China; 3 R&D Vision Sciences AMERA, Essilor International, Singapore, Singapore

**Keywords:** Refractive errors, Paediatric research

## Abstract

This study shows accommodative accuracy and distance accommodation facility in myopic children do not play a role in myopia progression. In 144 subjects, the monocular distance accommodative facility (DAF) and continuous accommodative stimulus–response curves (ASRCs) were measured at the enrolment. Retrospective and prospective refraction with regard to the enrolment visit were obtained from the outpatient database system based on noncycloplegic subjective spherical equivalent refraction (SER). The rate of myopic progression at enrolment was the first derivative of the Gompertz function, which was fitted with each subject's longitudinal refractive error data, including at least four records of SER with an interval of more than 6 months between each visit. A mixed linear model for multilevel repeated-measures data was used to explore the associations between the rate of myopia progression and accommodative parameters. The mean rate of myopia progression at enrolment was -0.61 ± 0.31 D/y with a mean age of 12.27 ± 1.61 years. By adjusting for age and SER, it was shown that the myopic progression rate was not associated with the accommodative lag (F = 0.269, P = 0.604), accommodative lag area (F = 0.086, P = 0.354), slope of ASRC (F = 0.711, P = 0.399), and DAF (F = 0.619, P = 0.432).

## Introduction

Myopia is the most common refractive error. The prevalence of myopia in young adolescents has increased substantially in recent decades, especially in East and Southeast Asia^1–3^. Rapid myopia progression might lead to high myopia with severe irreversible blinding ocular complications^4^, such as retinal detachment and glaucoma, affecting vision and quality of life. Although myopia has become a public health problem and has been studied for more than a century, the how or why of the onset and progression of myopia is unclear.

Retinal hyperopic defocus is regarded as a possible factor contributing to myopia and is considered the link between nearwork and myopia. Animal experiments^5,6^ have shown that the growth of the eye can be regulated by the sign of blur resulting from an additive optical lens. Hyperopic defocus induced by a negative lens accelerates axial length growth, while myopic defocus induced by a positive lens inhibits this growth. In humans, hyperopic defocus can be induced by accommodative lag caused by reduced accommodative response during nearwork. Thus, accommodative lag has been proposed to promote axial elongation^7–9^. Indeed, numerous streams of evidence have implied that accommodative lag is related to myopia. Accommodative lag has been reported to be higher in myopic individuals than in emmetropes^9,10^ at the same stimulus distance. Moreover, myopic children tend to read at a closer distance when compared to emmetropic children^11^, which may lead to increased lag of accommodation, as lag increases with accommodative demand^12^. Additionally, myopia progression in children was reported to be significantly greater with closer nearwork distances^13,14^ and slower with progressive addition lenses for children with higher lags of accommodation versus single-vision spectacles^15^. Therefore, it has been suggested that higher accommodative lag can induce faster eye growth in myopic children. Lan et al.^16^ conducted a longitudinal study of the association between myopia progression and accommodative lag at 33 cm in 62 myopic children (7–13 years). However, no association between accommodative lag and myopia progression was found, in accordance with the result of the CLEERE study^17^ on 592 myopic children. The lack of association may be due to accommodative stimuli adopted in previous studies. The reading distances of Chinese children have been shown to be very close, e.g., 16.3 ± 4.1 cm for Grade 2 for emmetropic children^18^, and highly task dependent^19^. Hence, the accommodative lag for a certain distance or stimulus (e.g., 3 D^16,20^ or 4 D^17^) need not be consistent with all reading conditions. This results in a lack of a relationship between accommodative lag and myopia in some studies but not in others. Another explanation can be that accommodative lag measurements used in prior studies were not sensitive enough. The area of the accommodative response function^7^, representative of the amount of accommodative response based on the linear regression of the accommodative stimulus–response function, was found to be highly related to myopia progression. Since accommodative lag is assumed to be a risk for myopia, the area of underaccommodation (the amount of accommodative lag) covering a range of stimuli might be a more sensitive index to predict myopia progression.

Other accommodative parameters have also been found to be associated with myopia. Reduced distance accommodative facility^21,22^ and shallower slope^10^ of the accommodative stimulus–response curve (ASRC) are more frequently observed in myopes than emmetropes. For their role in myopia progression, distance accommodative facility^20^ and the slope of ASRC had no significant effect^23^ on myopic young adults. However, to the best of our knowledge, no investigation has been conducted to assess the association between these accommodative parameters and myopia progression in children since accommodative characteristics were reported to change with age^24–26^.

The purpose of this investigation is to explore the relationship between different parameters of accommodation and myopia progression in Chinese myopic children.

## Results

One hundred forty-four subjects meeting the inclusion criteria were enrolled in this study. Thirty-one of the 144 subjects were excluded from the analyses because of their poor fit with R^2^ (less than 0.90 for the Gompertz function; 21 subjects) or lack of longitudinal data (less than 2 years; 10 subjects). The remaining 113 myopic children, consisting of 61 males and 52 females, were analysed. Table 1 shows the mean and range of each variable at enrolment.

**Table 1:** Mean and range of the variables at enrolment.

Variables	Minimum	Maximum	Mean ± SD
Age (years)	7.82	15.3	12.27 ± 1.61
Sphere (D)	− 6	− 0.75	− 2.99 ± 1.08
Cylinder (D)	− 1.5	0	− 0.34 ± 0.4
SER (D)	− 6.75	− 0.75	− 3.16 ± 1.13
Myopia progression (D/y)	− 1.42	0	− 0.61 ± 0.31
DAF (cpm)	2	45.5	18.29 ± 7.42
Slope of ASRC	0.81	1.2	1.02 ± 0.08
ALA 0–6 (D^2^)	3.25	11.85	6.84 ± 1.65
Accommodative lag at 0 D (D)	− 0.97	0.49	− 0.27 ± 0.3
Accommodative lag at 1 D (D)	− 0.13	1.35	0.51 ± 0.27
Accommodative lag at 2 D (D)	0.42	1.9	1.02 ± 0.28
Accommodative lag at 3 D (D)	0.64	2.21	1.33 ± 0.3
Accommodative lag at 4 D (D)	0.75	2.37	1.51 ± 0.33
Accommodative lag at 5 D (D)	0.75	2.52	1.61 ± 0.36
Accommodative lag at 6 D (D)	0.68	2.91	1.70 ± 0.4

*SER* spherical equivalent refraction, *DAF* distance accommodative facility, *slope of ASRC* the steepest slope of the accommodative stimulus–response curve, *ALA 0–6* the accommodative lag area between 0 and 6 D.

The longitudinal follow-up time, including retrospective and prospective visits, was 4.47 ± 1.33 years, ranging from 2.31 to 8.75 years. The Gompertz functions fit the refractive data well with a mean R^2^ value of 0.98 ± 0.02. The velocity of the refractive change in individual subjects at enrolment was the first derivative of the classic Gompertz function.

**Figure 1:**
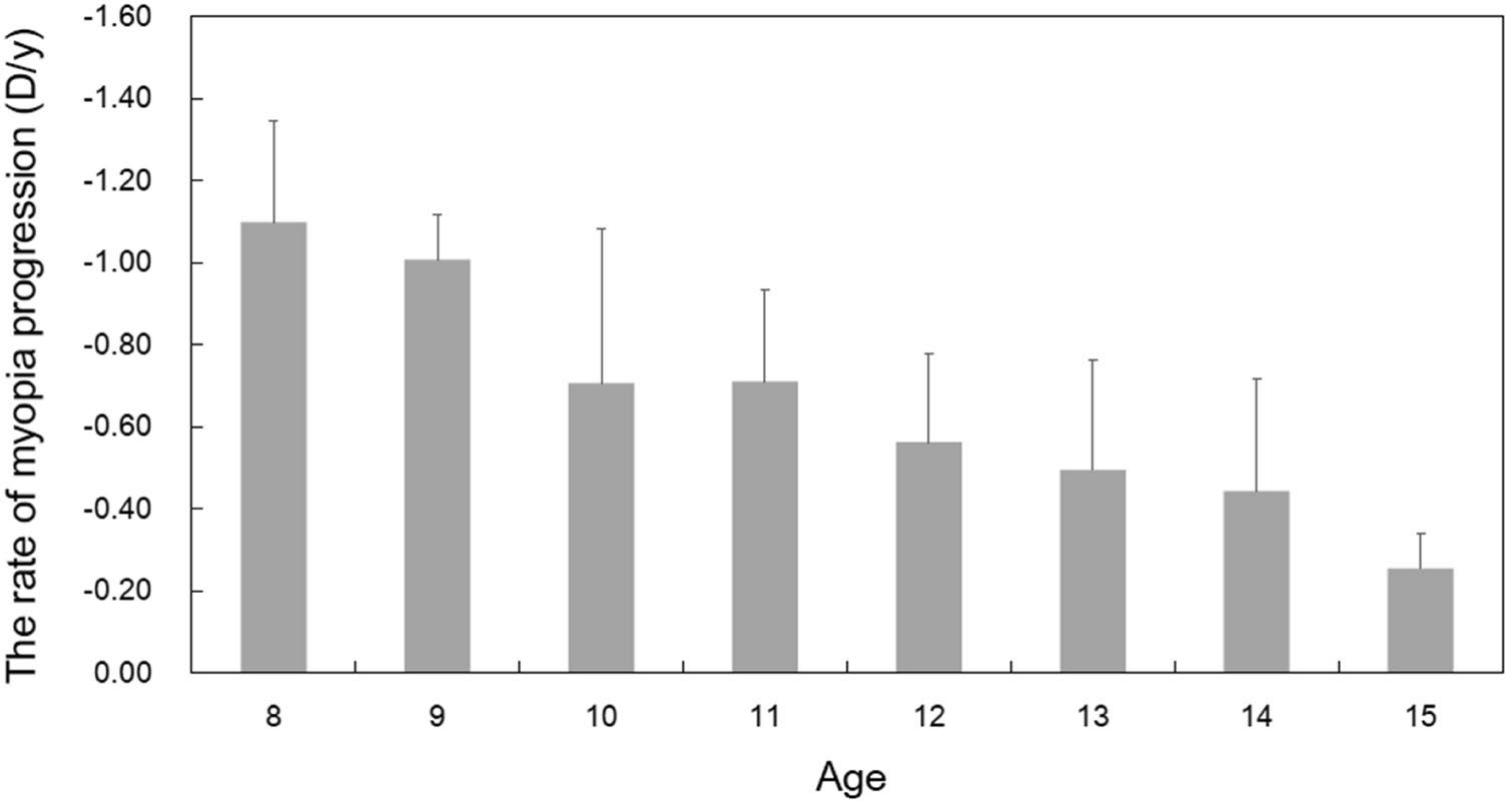
The rate of myopia progression at enrolment with age. The error bars represent the standard deviation.

The regression model revealed that age and SER were related to the myopia progression rate (Table 2). Myopia progression was shown as a function of age in Fig. 1. The rate of myopia progression at enrolment was − 1.10 ± 0.25 D/y at age 8 and then decreased gradually, reaching − 0.25 ± 0.08 D at age 15. The mean rate of myopia progression was − 0.61 ± 0.31 D/y for 113 subjects with a mean age of 12.27 ± 1.61 years. SER was significantly associated with myopia progression (F = 8.067, P = 0.005). The positive coefficient of the model (Table 2) indicated that children with higher myopia refractive error was associated with faster myopia progression, increasing by 0.021 D with each dioptre of SER (95% coefficient interval [CI]: 0.007 to 0.036).

**Table 2:** The coefficients of the mixed model regression analysis for myopia progression.

Parameter	Estimate	95% Confidence Interval		F	Sig
Intercept	− 1.744	− 2.022	− 1.467	178.803	< 0.001
Age	0.102	0.092	0.113	364.497	< 0.001**
SER	0.021	0.007	0.036	8.067	0.005*
**Different levels of stimulus**				0.030	1.000
Accommodative lag	− 0.017	− 0.082	0.048	0.269	0.604
ALA 0–6 (D^2^)	0.007	− 0.008	0.022	0.860	0.354
Slope of ASRC	− 0.104	− 0.344	0.137	0.711	0.399
DAF	0.001	− 0.001	0.003	0.619	0.432
Sex	0.012	− 0.020	0.044	0.519	0.471

*SER* spherical equivalent refraction, *DAF* distance accommodative facility, *slope of ASRC* the steepest slope of the accommodative stimulus–response curve, *ALA 0–6* the accommodative lag area between 0 and 6 D.

The mixed model regression analysis showed that accommodative lag was not associated with the rate of myopia progression at enrolment (F = 0.269, P = 0.604) adjusted by age and SER. The negative coefficient from the model (Table 2) indicated that myopia progression was affected by only 0.017 D for every D of lag (95% coefficient interval [CI]: − 0.082 to 0.048). The mean value of the accommodative lag area between 0 and 6 D was 6.84 ± 1.65 D^2^, and there was no significant association between the area and myopia progression (F = 0.086, P = 0.354); the coefficient value was small, at 0.007 (95% coefficient interval [CI]: − 0.008 to 0.022). The largest slope of the ASRC was 1.02 ± 0.08 and was not significantly associated with myopia progression (F = 0.711, P = 0.399); the coefficient value was -0.104 (95% coefficient interval [CI]: − 0.344 to 0.137).The DAF was 18.29 ± 7.42 cpm and unrelated to myopia progression (F = 0.619, P = 0.432); the coefficient value was − 0.001 (95% coefficient interval [CI]: − 0.001 to 0.003).

## Discussion

The purpose of present study was to investigate the association between accommodation and myopia progression of myopic children. It was found that myopia progression was not related to the accommodative lag at various proximities, accommodative lag area, slope of ASRC, or distance accommodative facility in enrolled subjects. The main outcome of our study was that although there was no significant association between accommodation and myopia progression, age and myopic refractive error were shown as predict factors.

In contrast to previous studies, we derived the myopia progression rate at enrolment from longitudinal datasets fitted with the Gompertz function. The high R^2^ value (mean value 0.98 ± 0.02) and low percentage of poor fit with R^2^ less than 0.90 (10.66% of subjects) supported that a double exponential function fits refractive data well during the myopization phase, in agreement with the findings of Thorn et al.^27^. The myopia progression rate decreased with age, which is consistent with the result of Donovan et al.^28^ for Asian children. The mean rate of myopia progression in our study (0.61 D) was slightly faster than the annual progression predicted by a quadratic equation at the same mean age in the study of Donovan et al. (0.48 D), probably due to the difference in calculation of the rate. Our result suggests that more attention should be given to younger myopes whose myopia progresses rapidly and who are more likely to become high myopes in later years. Myopia prevention strategies should therefore be started from an early age to decrease the prevalence of high myopia^29^.

The present study showed that children with higher levels of myopia were likely to have slightly faster progression, in accordance with previous studies^14,30–32^. It is possible that more relative hyperopic peripheral defocus associated with higher myopia^33^ leads to faster myopia progression^34^. In addition, a higher-order aberration^35^ in the more myopic eye might stimulate eye growth^36^.

For a few decades, accommodative lag has been considered a putative risk for the development of myopia. In this study, the accommodative response was measured using a Badal stimulator under which blur is the sole cue for accommodation. For a stimulus proximity of 4D, the average value of the lag of accommodation was 1.51 ± 0.33 D, comparable to the results of Berntsen et al.^17^ (1.51 ± 0.63 D) with similar accommodative testing conditions. The assumed relationship between accommodative lag at various proximities with myopia progression could not be identified in this study. Furthermore, the accommodative lag area between 0 and 6 D cannot predict myopia progression in our study. According to our data and in accordance with previous studies^16,17^, there is no clear evidence that accommodative lag is a risk for myopia progression, implying that the contribution of accommodation-induced hyperopic defocus in the development of myopia in myopic children may be negligible even with a high amount of defocus at a closer distance. One hypothesis explaining these results is that the mechanisms of myopia onset and myopia progression are different. Myopia progression is not regulated by accommodative lag in humans, despite the strong evidence that hyperopic defocus stimulated by optical lenses can induce myopia in animals. Lu et al.^37^ found that myopia developed rapidly within 2 days of negative-lens wear and slowly afterwards, then did not progress after 10 days in guinea pigs, supporting the hypothesis. Another possible explanation for this is that accommodative lag is a consequence of myopia^38^, rather than a cause. The negative result provides support for the observations that although the intensity of nearwork increases with age^39^, myopia progression does not increase with age. This finding also supports that multifocal lenses designed to reduce accommodative lag to retard myopia progression have a modest clinical significance^40,41^.

The slope of the ASRC in our study (1.02 ± 0.08) was steeper than that previously published by Gwiazda et al.^10^ (0.78) in myopic children. This could be because the steepest slope of the ASRC, instead of the gradient of the linear regression, was adopted in our study. However, the slope was higher than that for young adults (0.80) with the same method and calculation in our previous study^42^. This discrepancy may be due to a larger accommodative response in younger subjects^24^. The present study reveals that the slope of the ASRC is not linked to myopia progression in children, in agreement with the result of young adults^23^, although this slope was reduced in progressing myopes but not in stable myopes^8^. Hence, the gradient of accommodative response appears to be mainly an index that reflects the accuracy of accommodation but not a risk factor for myopia progression.

As an indicator of accommodative dynamics, DAF was found to be reduced both in myopic children^21^ and myopic young adults^22^ compared with emmetropes, possibly because of the lower accommodative response to negative lenses^10^ and the lower velocity of accommodation and disaccommodation^43^. Similar to Allen and O'Leary^20^, who studied young adults, we did not find any association between myopia progression and DAF. This result is also consistent with other research^44,45^ that found that visual training to improve accommodative dynamics failed to retard myopia progression, implying that accommodative dynamics are not a factor for myopia progression.

This study has several limitations. First, myopia progression in our sample was derived from retrospective data in the outpatient system, and the time intervals between visits were uneven. However, we calculated the rate of myopia progression at enrolment from the fitting curve, compensating for this shortcoming to a certain extent. Second, our study fitted the Gompertz function with subjective refractions instead of cycloplegic autorefraction, which is considered the gold standard for determining refractive status^46,47^. However, it would only have a negligible effect on the Gompertz fit and the results of the myopia progression rate. Moreover, it was reported that subjective refraction was comparable to cycloplegic refraction in myopes^48^. Third, no information about other known risk factors could contribute to myopia progression, such as the time of near work or outdoor activity. It would have been more convincing to combine different accommodative lags with the time of nearwork, which represents the accumulated effect of hyperopic defocus.

In summary, a key finding of the present study was that accommodative accuracy and distance accommodative facility in myopic children were not associated with myopia progression in our sample. Myopization is a complex, multifactorial process that involves factors other than accommodation.

## Methods

One hundred forty-four children from the Primary Care Department of the Eye Hospital affiliated with Wenzhou Medical University were enrolled between August 2014 and September 2015. Subjects meeting the following criteria were recruited: available retrospective data for more than 6 months prior to the enrolment from the outpatient system; aged 8 to 15 years at the time of enrolment; spherical equivalent of more than − 0.75 D in each eye, astigmatism less than 2.00 D in either eye, anisometropia less than 1.00 D, monocular best visual acuity better than 0.1 of logMAR; no strabismus by cover test with distance-corrected spectacle; no progressive addition lens or contact lens wearing history; and no history of any ocular disease. The study conformed to the tenets of the Declaration of Helsinki and was approved by the Ethical Committee of the Eye Hospital affiliated with Wenzhou Medical University. Informed consent was obtained from the subjects and their legal guardian.

### Procedure

The subjects were measured by following procedures on the enrolled day.

### Refraction

Retinoscopy was performed before subjective refraction to exclude subjects with greater fluctuations in accommodation, or with more than 0.50D-discrepancy between retinoscopy and subjective refraction. Subjective refraction was performed according to the principle of "maximal positive lens maximal visual acuity" to avoid overcorrection. Data were collected by an experienced optometrist.

### Distance accommodative facility (DAF)

DAF was measured for the right eye, which fixated at the central letter in a 3 × 3 array of 20/30 letters placed 4.5 m away, while the left eye was occluded. A − 2.00 D trial lens was placed in the trial frame in front of the eyes, which made the letters blurred for a short time, and then the letters became clear again. As soon as the letters were clear, the subject informed the examiner. The examiner then removed the lens, which made the letters blurred again for a short duration, and the subject informed the examiner as soon as the letters could be seen clearly again. A full cycle consisted of two phases, i.e., with and without − 2.00 D. The number of cycles completed in 1 min was recorded. The subjects were given a guidance before starting the measurements to ensure that they understood the procedures. One-minute breaking was required between guidance and measurement to avoid the aftereffect of accommodation.

### ASRC measurements

The ASRC method was explained in a previous study^42^. The motorized Badal system consisted of a Badal lens with a power of + 5.75 D and a moving auxiliary lens with a power of + 3.33 D mounted on a Grand Seiko autorefractor (WAM-5500; Grand Seiko Co., Ltd., Japan) to produce a wide range of accommodative stimuli from − 1.61 D to + 14.83 D with a speed of 0.40 D/s. Accommodative response was measured with the WAM-5500 in high-speed mode at a sampling speed of 5 Hz. A computer program initiated the motor system of the auxiliary lens and synchronized its position with the measurements of the WAM-5500. The right eye viewed through the motorized Badal system, while the left eye was occluded. Any refractive error was fully corrected by trial lenses. The fixation target set at 4.5 m was a 3 × 3 array of 20/30 high-contrast letters^49^ printed on a white background with a mean luminance of 18 cd m^−2^. The subjects were instructed to keep the target as clear as possible and to fixate the target even if it became blurred or unresolvable.

**Figure 2:**
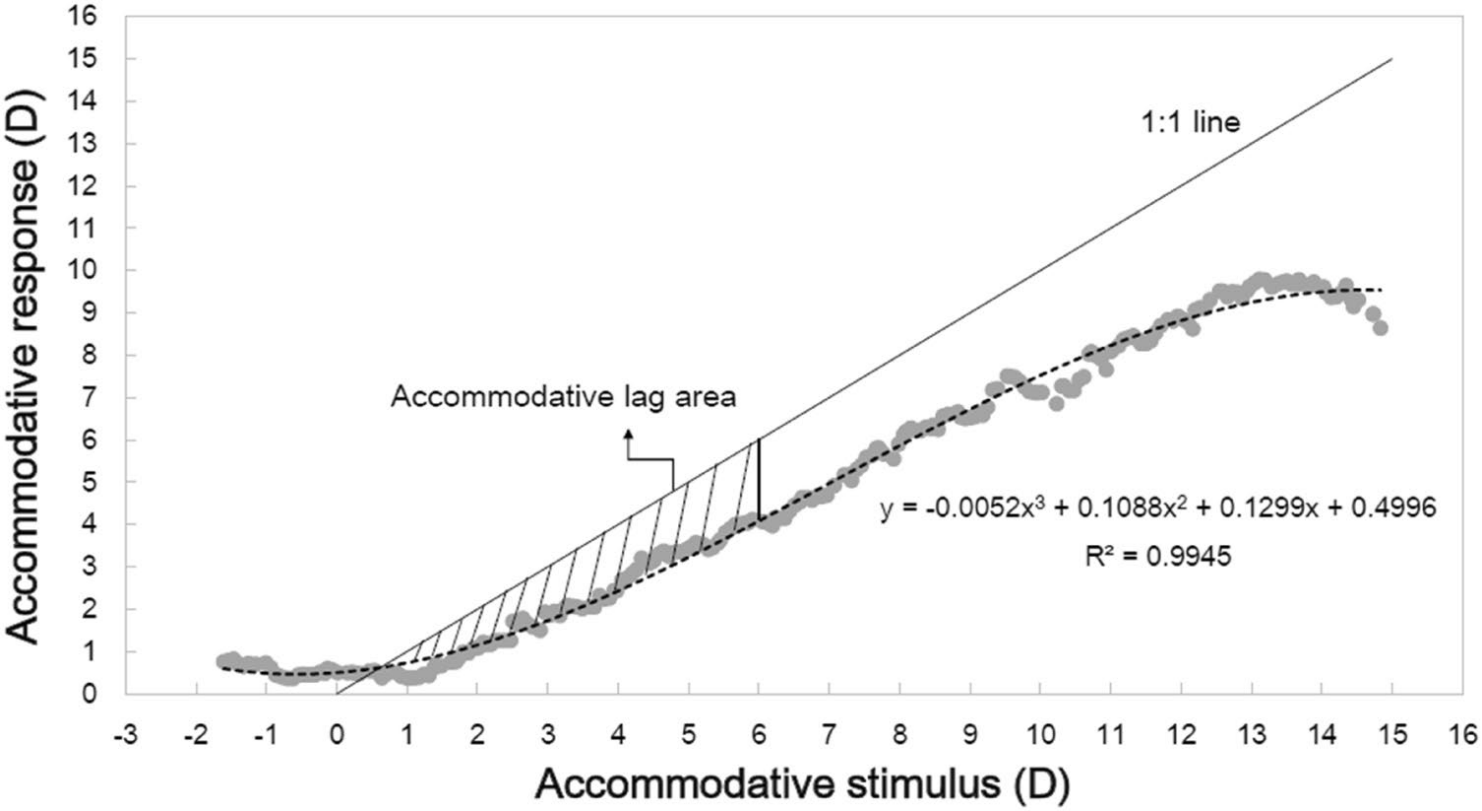
Accommodative stimulus–response curve and accommodative lag area. ASRCs were fitted with a 3-degree polynomial equation (dashed line). The shaded area represents the accommodative lag area between the 1:1 line and the fitted accommodative stimulus–response curve between 0 and 6 D.

The accommodative stimulus and accommodative response were adjusted at the corneal plane^50^. Continuous accommodative responses were fitted with a 3-degree polynomial equation as a function of accommodative stimulus. The steepest slope of the curve was calculated. Accommodative lag area was defined as the area of underaccommodation between the 1:1 line and the response curve in the stimulus–response plot between 0 and 6 D (ALA 0–6) (see Fig. 2). The accommodative lag for a given stimulus was obtained by subtracting the accommodative response from the accommodative stimulus. The ASRC of each subject was measured at least three times, and the curve with the best fit (highest R^2^ value) was chosen for analysis.

### Myopia progression at enrolment

**Figure 3:**
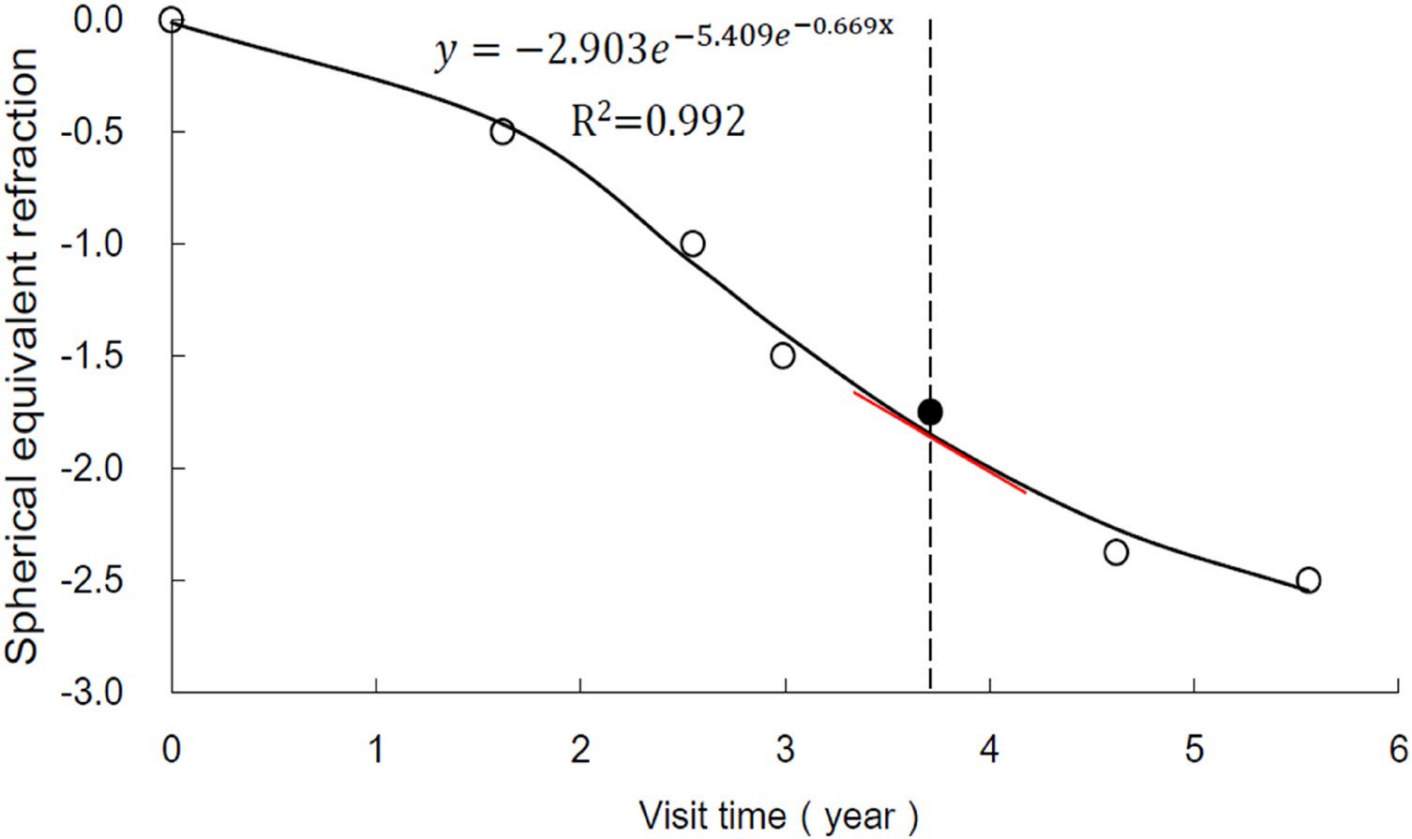
The trend in myopia progression of one subject fitted with the Gompertz function (solid line). The filled circle represents the SER at enrolment, while open circles represent the retrospective and prospective SER. The slope of the tangential line (red line) at enrolment is the first derivative of the Gompertz function (dashed line), which represents the rate of myopic progression at enrolment.

In this study, retrospective and prospective refraction from the enrolment could be obtained from the outpatient database system based on noncycloplegic subjective spherical equivalent refraction (SER). Because the development of myopia is nonlinear^27^, the classic Gompertz function is fitted with each subject's longitudinal dataset, including at least four records of SER with an interval of more than 6 months between each visit (Fig. 3). The equation is $Y=a{e}^{-b{e}^{-kx}}$, where Y equals the refractive error at a given visit and x is the visit time relative to the visit that obtained the first record of SER. Myopia progression rate at enrolment is the first derivative of the Gompertz function, defined as the change in SER per year (Fig. 1).

### Statistical analysis

Data from the right eye were analysed by IBM SPSS Statistics software, version 25 (SPSS, Inc., Chicago, IL). A nonlinear regression analysis was used to fit the refractive data with the Gompertz function. The goodness of curve fit depended on the R^2^ value. A mixed linear model for multilevel repeated-measures data was used to explore the associations between the rate of myopia progression at enrolment and accommodative parameters adjusted by age and SER. The covariates evaluated in the models were the accommodative lag at different stimuli, largest slope of ASRC, accommodative lag area, DAF and sex.
